# Respiratory Efficacy of a Multivalent Marker Vaccine Against Bovine Viral Diarrhoea Virus Types 1 and 2, Infectious Bovine Rhinotracheitis Virus, Bovine Respiratory Syncytial Virus, and Bovine Parainfluenza-3 Virus in Young Calves

**DOI:** 10.3390/vaccines13100999

**Published:** 2025-09-24

**Authors:** Carlos Montbrau, Marta Gibert, Marina Solé, Isabel Barril, Mercè Roca, Lucia Acal, Berta Vázquez, Joaquim Mallorqui, Ricard March

**Affiliations:** 1 Hipra Scientific, S.L.U., Avinguda La Selva 135, 17170 Amer, Spain; marta.gibert@hipra.com (M.G.); marina.sole@hipra.com (M.S.); isabel.barril@hipra.com (I.B.); merce.roca@hipra.com (M.R.); lucia.acal@hipra.com (L.A.); joaquim.mallorqui@hipra.com (J.M.); ricard.march@hipra.com (R.M.); 2 HIPRA S.A., Avinguda La Selva 135, 17170 Amer, Spain; berta.vazquez@hipra.com

**Keywords:** bovine respiratory disease (BRD), bovine viral diarrhoea virus (BVDV), infectious bovine rhinotracheitis virus (IBR), bovine respiratory syncytial virus (BRSV), bovine parainfluenza-3 virus (PI-3), vaccine, bovine herpesvirus type 1 (BoHV-1), experimental infection

## Abstract

**Background/Objectives**: A new multivalent vaccine (DIVENCE^®^ PENTA), containing Bovine viral diarrhoea virus (BVDV) types 1 and 2 recombinant proteins, live gE/tk double gene deleted Bovine Herpesvirus type 1 (BoHV-1 or IBR), live attenuated Bovine respiratory syncytial virus (BRSV) and inactivated parainfluenza-3 virus (PI-3) has been designed to protect cattle against the main viral pathogens associated with Bovine respiratory disease (BRD). The aim of this study was to demonstrate the efficacy of DIVENCE^®^ PENTA against experimental infections with BVDV-1, BVDV-2, IBR, BRSV and PI-3 in young calves. **Methods**: Ten-week-old calves were given two intramuscular doses three weeks apart. The efficacy was evaluated by means of an experimental challenge three weeks after vaccination. Serology, clinical signs, rectal temperature, white blood cell count, viral shedding and lung lesions were monitored after the challenge. **Results/Conclusions**: The results demonstrated a significant sparing of BRD in calves vaccinated with DIVENCE^®^ PENTA, as evidenced by fewer clinical signs, lower rectal temperatures, reduced viral shedding and less severe pulmonary lesions compared to control animals. A significant reduction in hyperthermia, leukopenia and viraemia post-challenge was also observed, highlighting the efficacy of the multivalent vaccine against BVDV types 1 and 2, IBR, BRSV and PI-3 in young calves.

## 1. Introduction

Bovine respiratory disease (BRD) is a multifactorial syndrome caused by a combination of viral and bacterial pathogens, along with environmental risk factors, that leads to the development of respiratory tract infections, such as bronchopneumonia or pleuropneumonia [1,2]. Common clinical signs of BRD include tachypnoea, dyspnoea, mucopurulent nasal discharge, cough, and elevated temperature [3]. The disease is associated with high morbidity and mortality in young calves, resulting in both short-term and long-term economic losses [2,4]. The main viral infectious agents associated with BRD include bovine respiratory syncytial virus (BRSV), bovine herpesvirus type 1 (BoHV-1), bovine rhinotracheitis virus (IBR), bovine viral diarrhoea virus (BVD) and to a lesser extend parainfluenza-3 virus (PI-3). All these viruses are known to contribute to the pathogenesis of BRD, either individually or in combination with each other, potentially aggravating the severity of outbreaks. Furthermore, they may interact with bacterial pathogens such as *Mannheimia haemolytica*, *Pasteurella multocida* and *Histophilus somni* [5,6,7,8].

Given the multifactorial nature of BRD and the growing concerns about antimicrobial resistance, there is an increasing emphasis on preventive strategies that prioritise immunity over antibiotic use. Accordingly, the prophylactic administration of antibiotics and other medicinal products in livestock has been strictly regulated and banned in Europe since 2022, with metaphylactic use now restricted to cases where the risk of infection is exceedingly high [9]. Under this scenario, the optimisation of both individual and herd immunity while reducing exposure to the BRD pathogens, is fundamental for effective prevention and control of the disease [10]. Notably, multiviral BRD vaccines have demonstrated strong serological responses in calves, highlighting their value in conferring immunity against multiple pathogens [11,12]. Therefore, the implementation of vaccination programmes covering the most significant respiratory viruses along with environmental and management improvements is essential [7].

To achieve broad, effective protection against BRD, vaccination strategies must address the multifactorial nature of the syndrome, and the diversity of the pathogens involved. A wide range of administration routes and effective vaccines against BRD are available on the market, including modified-live (MLV), inactivated, and recombinant formulations. A foundational step in this approach is the early intranasal administration of MLV BRSV vaccines. This proceeding has demonstrated the ability to elicit a strong mucosal IgA response even in the presence of maternal antibodies [13,14]. As a result, a widespread practice is the intranasal live vaccination of young calves to provide early immunity in replacement animals or prior to their arrival at feedlots, during the major period of incidence [13].

Building on this initial protection, broader immunization is achieved via the use of multivalent vaccines that target additional key respiratory viruses. Among these, marker vaccines, particularly IBR MLV gE-marker vaccines, have proven effective in reducing culling rates and preventing seroconversion in naïve animals [15]. In the European context, the addition of BoHV-1 in vaccination programs requires the use of a monovalent marker vaccine requiring the prescription of at least two separate vaccines to ensure comprehensive coverage against the most relevant viral agents (Commission Delegated Regulation (EU) 2020/689 [16]). This layered vaccination strategy underscores the importance of both early mucosal priming and subsequent systemic wide protection to mitigate the impact of BRD in cattle populations [7].

Recently, a new multivalent viral vaccine, DIVENCE^®^ PENTA, has been developed to protect cattle from the primary viral pathogens linked to BRD. This vaccine combines recombinant proteins for BVDV-1 and BVDV-2, live gene-deleted BoHV-1 (gE/tk double deletion), live-attenuated BRSV and inactivated PI-3. Its effectiveness in reducing the incidence, morbidity, and need for BRD treatments has been demonstrated in a randomized field trial conducted in commercial fattening units [17]. The vaccine also provides foetal protection in animals exposed to BVDV-1 and BVDV-2 [18], and cross-protection to several BVDV subtypes [19]. However, the vaccine’s efficacy against the role of these viruses during a BRD outbreak in an experimental challenge has not yet been reported. The purpose of the study was, therefore, to determine the efficacy of the DIVENCE^®^ PENTA vaccine, administrated intramuscularly in 10-week-old calves, against experimental infections with BVDV types 1 and 2, IBR, BRSV and PI-3.

## 2. Materials and Methods

### 2.1. Study Design

**Figure 1:**
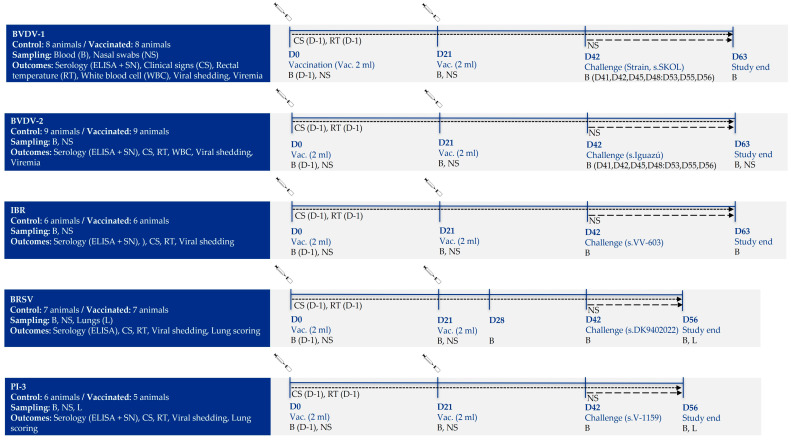
Experimental design of the study.

Five different pre-clinical trials were conducted to demonstrate the efficacy of DIVENCE^®^ PENTA for each viral antigen: BVDV-1, BVDV-2, IBR, BRSV and PI-3. The seventy-one 10-week-old calves (average age of 70 days: ranging from 54 to 117 days old) were divided into those 5 different trials and allocated into two groups per trial–a vaccinated and control group (Figure 1). A total of 16 calves were enrolled in the BVDV-1 trial. None of these calves had any antibodies against BVDV and no virus neutralizing antibodies against BVDV-1 were detected. Eight animals were vaccinated with DIVENCE^®^ PENTA following the procedure described above, whereas PBS was administered to the other eight calves used as a control group. Three weeks after the second dose of DIVENCE^®^ PENTA or PBS, all animals were intranasally challenged using the virulent BVDV-1 strain SKOL. The challenge was conducted by spraying 5 mL of virus inoculum into each nostril and applying 1.6 × 10^7^ TCID_50_ of challenge virus per animal. For the BVDV-2 trial, 18 calves were randomly distributed into two groups of nine animals, in which one group was vaccinated with the DIVENCE^®^ PENTA vaccine and the other group received PBS. Prior to vaccination, none of these calves had any antibodies against BVDV nor virus neutralizing antibodies against BVDV-2. On day 21 post-vaccination, all animals were intranasally challenged by spraying 5 mL per nostril of the virulent BVDV-2 strain Iguazú. Each animal received 6.3 × 10^4^ TCID_50_ of BVDV-2 challenge virus.

A total of twelve calves with no antibodies against IBR or virus neutralizing antibodies against IBR were enrolled in the IBR trial. Six of those calves were vaccinated using DIVENCE^®^ PENTA and the remaining six calves (control group) received PBS. Three weeks post-vaccination, the calves were intranasally challenged by spraying 1 mL per nostril of the virulent VV-603 IBR strain. Each animal received 1 × 10^7^ TCID_50_ of IBR challenge virus.

For the BRSV trial, 14 calves were randomly distributed into two groups of seven, one group vaccinated with the DIVENCE^®^ PENTA vaccine, and the other group received PBS. All animals were free of antibodies before vaccination. All calves enrolled in the BRSV trial were experimentally infected 21 days post-vaccination with the BRSV DK9402022 strain. The infection was conducted by aerosol exposure to approximately 100 mL (103.46 CCID_50_/mL) of BRSV in a sealed 7 × 2.5 × 2 m. transport (stock) trailer (35 m^3^ of air space) for approximately 40 min and afterward maintained as a single group in one pen. For aerosol delivery, 100 mL of the inoculum was used in an ultrasonic nebulizer (U17, Omron^®^) placed equidistant approximately 1.5 m off the floor of the trailer.

Finally, for the PI-3 trial, eleven calves were randomly distributed into a vaccinated (n = 6) and a control group (n = 5). Three weeks after vaccination, all animals were intranasally infected, spraying 5 mL per nostril of the virulent V-1159 PI-3 strain. Each animal received 1 × 10^8^ TCID_50_ of PI-3 challenge virus.

### 2.2. Animals and Housing

A total of 71 ten-week-old calves were enrolled in this study. These animals were sampled before the study to assess antibodies against BVDV, IBR, BRSV and PI-3 by ELISA, as well as neutralizing antibodies in sera (SN) against BVDV-1, BVDV-2, IBR and PI-3. All these animals were confirmed to be free of persistent BVDV infection by real-time reverse transcription polymerase chain reaction (RT-qPCR). All calves were housed in pens outdoors during the vaccination phase, from the beginning of the study until two days prior to the challenge. During the challenge phase, from two days before up to day 14 (for BRSV and PI-3 trials) or day 21 (for BVDV and IBR trials) after the experimental challenge, all animals were grouped in two mixed pens per study.

### 2.3. Vaccination

The multivalent vaccine, DIVENCE^®^ PENTA was given intramuscularly in two doses (2 mL dose) three weeks apart starting at 10 weeks of age. The BVDV antigens in the vaccine serial were batched at their minimum protective dose (MPD) level. In all trials, control animals received 2 mL of phosphate-buffered saline solution (PBS) administered following the same procedure described in the vaccinated group.

### 2.4. Challenge Viruses

The challenges were performed using strains isolated from Europe and Americas. The virulent non-cytopathic BVDV type 1b strain SKOL and the non-cytopathic BVDV type 2 strain Iguazú were used as challenge viruses for BVDV. Both strains were isolated from aborted foetuses in field cases. The virulent VV-603 IBR strain and the V-1159 PI-3 strain were used to challenge calves in the IBR and PI-3 clinical trials, respectively. Both strains were isolated from field cases. The challenge inoculum for the BRSV study consisted of a bronchoalveolar wash obtained from two newborn calves infected with BRSV, using the Strain DK9402022. The BRSV lung lavage fluids were confirmed to be free of bacterial contamination, *Mycoplasma* spp., *Mannheimia* spp., *Histophilus somni* and *Pasteurella multocida*, as well as negative for IBR, PI-3, and BVDV. The virus vials were stored at −70 °C ± 10 °C and at the day of the challenge were thawed at 37 °C. No dilution was needed.

### 2.5. Clinical Assessment

All calves were monitored to assess general clinical signs throughout the entire study. Furthermore, respiratory clinical signs and rectal temperatures were recorded on days −1 and 0, before challenge, and from 1 to 21 days after challenge for BVDV-1, BVDV-2 and IBR, and from 1 to 14 days after challenge for BRSV and PI-3, following the recommendations of the European Pharmacopoeia. Clinical assessments were conducted each morning at the same time by personnel blinded to the treatment allocation in each trial. Clinical signs monitored included depression, dyspnoea, nasal and ocular discharges, coughing and diarrhoea as described by Taberner et al. [18]. At the end of the BRSV and PI-3 trials, the respiratory tract of each calf was collected and analysed to determine the percentage of pneumonic tissue in each lung lobe to calculate the total percentage of pneumonic lung lesions, as reported previously by other authors [20].

### 2.6. Sample Collection

All calves enrolled in the different clinical trials were sampled to obtain sera samples the day previous to vaccination or the day of the first and second vaccination (day −1 of study, for BVDV trials or day 0 for BRSV, PI-3 and IBR trials and 21 of study), the day of challenge (day 42 of study), and at the end of the challenge period (day 56 for BRSV and PI-3 trials, day 63 for BVDV-1, BVDV-2 and IBR trials). Additionally, in the BRSV trial, a serum sample was collected 7 days after the second vaccination (day 28). Nasal swabs were also collected from both nostrils at days 0 and 21 (first and second vaccination days), and each day from the day of challenge (day 42 of study) to the end of the challenge (day 56 for BRSV and PI-3 trials, day 63 for BVDV-1, BVDV-2 and IBR trials). For the BVDV and IBR trials, swabs were placed in 3 mL of MEM Glasgow medium. For the BRSV trial, swabs were placed in 5 mL PBS containing antibiotic. For the PI-3 trial, swabs were stored in 2 mL of PBS containing antibiotic. All swabs were stored at −70 °C or below until they were cultured for quantitative virus isolation. For the BVDV trials, blood samples for white blood cell (WBC) counts were collected in EDTA tubes one day before the challenge, the day of challenge, and on Days 3, 6, 7, 8, 9, 10, 11, 13 and 14 post-challenge. In the BRSV trial, lobe swabs in PBS were also collected from those lobes in which lesions were observed.

### 2.7. Sample Testing

ELISA antibodies against BVDV, IBR, BRSV and PI-3 were determined by use of commercial ELISA kits according to the manufacturers’ instructions, which provide qualitative or semiquantitative evidence of specific antibody presence without distinguishing antibody isotypes. For BVDV, a cut-off sample to positive ratio (S/P) value ≥ 0.30 was considered positive (BVDV Total antibody test, IDEXX). For IBR, a cut-off IRPC value ≥ 15 was considered positive (CIVTEST BOVIS antibody test, HIPRA). For BRSV, the cut-off OD value was calculated for each plate following manufacturer instructions, cut-off = NC − [(NC − PC) × 0.4] and all samples with OD higher than cut-off were considered negative (INgezim BRSV Compac R.12.BRS.K3, INGENASA). And for PI-3, a cut-off S/P% value ≥ 20 was considered positive (PI-3 virus antibody test kit, IDEXX). The antibody ELISA titre for BRSV and PI-3 was defined following the manufacturer instructions. Serum neutralizing (SN) antibody titres were measured using a standard serum neutralization assay, in which serial serum dilutions are incubated with a constant dose of virus and then assessed for their ability to inhibit viral replication in cell culture, thereby providing a functional measure of neutralizing capacity. BVDV-1 and BVDV2 SN were measured using a standard serum neutralization assay as described by Montbrau et al. [19]. SN in GBK and MDBK cells in 96-well plates was performed to quantitate SN antibodies against IBR and PI-3, respectively. A constant virus titre (10^2.5^ CCID_50_ for IBR and 10^2^ CCID_50_ for PI-3) was incubated with two-fold dilutions of sera. Culture plates were incubated for seven days and visually assessed for a virus-induced cytopathic effect. WBC counts for the BVDV trials were analysed using a semi-automated electronic cell counting device (XN-1000 Sysmex, Laboratorio Echevarne, Barcelona, Spain), as described by Taberner et al. [18]. To evaluate virus detection, a RT-qPCR assay from buffy coats was conducted for the BVDV trials, as described by other authors [18] and from nasal swabs for the BRSV trial using primer pairs previously described [13]; moreover, a cell culture assay to detect the virus in the nasal swabs was performed for the IBR and PI-3 trials. For IBR and PI-3 titration assays, nasal swab samples were inoculated in duplicate into 96-well plates that were seeded with GBK and MDBK cells, respectively, and incubated for four days (37 °C, 5% CO_2_). After incubation, an immunoperoxidase monolayer assay (IPMA) was performed, as described by Taberner et al. [18], using IBR and PI-3 specific monoclonal antibodies.

### 2.8. Statistical Analysis

In each trial, the two treatment groups (vaccinated and control) were analysed and compared based on primary parameters, including serology (ELISA and SN antibodies), rectal temperature, clinical signs, WBC count (for BVDV trials only) and virus shedding, using the R Software (version 4.4.0) and Microsoft^®^ Excel 2010 (Microsoft corp.). When required, data were transformed (i.e., Log2 SN Titre, Log-WBC, Log10 CCID_50_/mL IBR and PI-3, Log2-Total virus BRSV) to satisfy assumptions of normality. *p* < 0.05 was considered the limit for statistical significance.

A *t* test was used to compare daily rectal temperatures, average rectal temperatures during the peak period (days six to nine post-challenge), WBC counts per day, the average WBC counts after challenge, and the average clinical signs score after challenge between groups. Fisher’s exact test was used to compare the percentage of animals with hyperthermia (>39.5 °C; [21]), the proportion of animals with positive ELISA and SN antibodies, and the percentage of samples with shedding per day between groups. The Mann–Whitney U test was used to compare the average number of days with hyperthermia, respiratory clinical signs scores at each time point, ELISA or SN antibody levels per sample day, and the percentage of total lung lesions (BRSV and PI-3 trial) between groups. In addition, the daily average titration, the total virus shedding post-challenge, the duration of days detecting the virus per group, the differences between basal temperature (average of day −1 and day 0) and the day with the maximum temperature peak within each group, as well as the differences between the basal WBC count (average of day −1 and day 0) and the day with the greatest decrease in WBC counts within each group were compared using a *t* test, ANOVA or Mann–Whitney U test, according to normality of the data in each of those trials.

## 3. Results

### 3.1. Efficacy Against BVDV-1 Challenge

**Figure 2:**
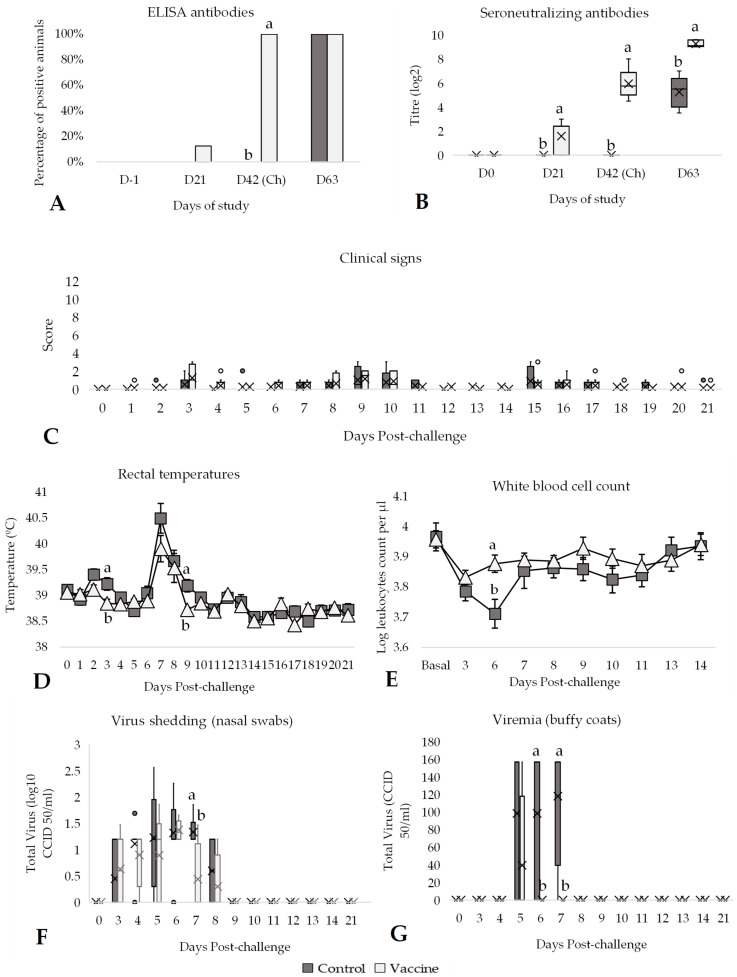
Efficacy of the DIVENCE^®^ PENTA vaccine against a BVDV-1 challenge. Control: 8 animals, Vaccinated: 8 animals. (**A**) Percentage of animals with positive ELISA antibodies per group from day 0 to 63 of study (bars; Fisher’s exact test). (**B**) Serum neutralising antibodies per group measured by serum neutralisation assay from day 0 to 63 of study (boxplot log2 values; Mann–Whitney U test). (**C**) Daily scoring of clinical signs per group from day of challenge until 21 days post-challenge (boxplot; Mann–Whitney U test). (**D**) Daily rectal temperatures per group from day of challenge until 21 days after challenge (mean ± SE; *t* test). (**E**) White blood cells count per group from basal (average of day −1 and day of challenge) until 14 days after challenge (log mean ± SE; *t* test). (**F**) Virus shedding per group detected in nasal swabs by PCR and percentage of animals shedding from day of challenge until 21 days after challenge (boxplot log10 values; Mann–Whitney U test). (**G**) Viraemia per group detected in buffy coats by PCR from day of challenge until 21 days after challenge (boxplot; Mann–Whitney U test). ^a,b^ Different superscripts indicate statistical differences between control and vaccinated groups (*p* < 0.05). Ch, challenge.

At day 42 of the study (day of challenge), 100% of vaccinated animals were seropositive by both ELISA and SN tests, while all control animals remained negative until challenge (Figure 2A,B). Actually, vaccinated animals had a significantly higher (*p* < 0.05) average of antibody titres (SN antibodies) compared to control animals (Figure 2B). After challenge, all animals of both groups presented antibodies against BVDV, but the average antibody titres (SN antibodies) in the vaccinated group were significantly higher (*p* < 0.05) than in the control group (Figure 2B). Moreover, no relevant adverse systemic effects or injection site reactions were observed during the vaccination phase. No differences between groups were observed regarding post-challenge clinical signs (Figure 2C, Supplementary Table S1).

The rectal temperatures of the animals were also measured from the day of challenge until 21 days after challenge (day 63 of study). On the challenge day, all the animals enrolled in the study had temperatures below 39.5 °C (Figure 2D). After the challenge, a first mild peak of rectal temperatures was observed on days 2 and 3. A second peak of rectal temperatures was also seen on days 7, 8 and 9 post-challenge. The average of rectal temperatures in the control group was significantly higher (*p* < 0.05) than in the vaccinated group on days 3 and 9 post-challenge (Figure 2D). During the second peak, the average of rectal temperatures from day 6 to day 9 in the control group was significantly (*p* < 0.05) higher (39.60 ± 0.09 °C) than in the vaccinated group (39.27 ± 0.10 °C; Supplementary Table S1). Consequently, the number of days with hyperthermia (>39.5 °C) was significantly higher (*p* < 0.05) in the control group (2.5 days) compared to the vaccinated group (1.13 days) (Supplementary Table S1). In addition, a clear decrease in leukocytes was observed in the control group after challenge and the average WBC count was significantly lower (*p* < 0.05) compared with the vaccinated group on day 6 post-challenge (Figure 2E). Furthermore, a significant reduction in WBC counts between the basal (average of one day before challenge and the challenge day) and day 6 post-challenge was observed in the control group (*p* < 0.01), whereas no significant change was detected in the vaccinated group over the same period (*p* = 0.830). Nasal swab and buffy coat samples were collected from challenged calves from day 0 of the study to day 21 post-challenge for detection of virus shedding. All the samples during the vaccination period until challenge day were negative by PCR. After challenge, the virus shedding, viraemia and the percentage of positive samples were significantly higher (*p* < 0.05) in the control group compared to the vaccinated group on day 7 post-challenge (Figure 2F,G). For nasal swab samples, seven days after challenge, 100% of the animals from the control group were shedding BVDV-1 virus, whereas in the vaccinated group, only 38% of the animals were excreting virus (Figure 2F). Additionally, the control animals were positive to buffy coats samples for significantly (*p* < 0.05) higher days (2.00 days) compared to the vaccinated group (0.25 days) post-challenge (Supplementary Table S1). Considering the entire post-challenge period, the average of viraemia (total virus) from buffy coats was also significantly higher (*p* < 0.05) in the control group (62.80 ± 5.54 CCID_50_/mL) compared to the vaccinated group (7.85 ± 1.71 CCID_50_/mL; Supplementary Table S1).

### 3.2. Efficacy Against BVDV-2 Challenge

**Figure 3:**
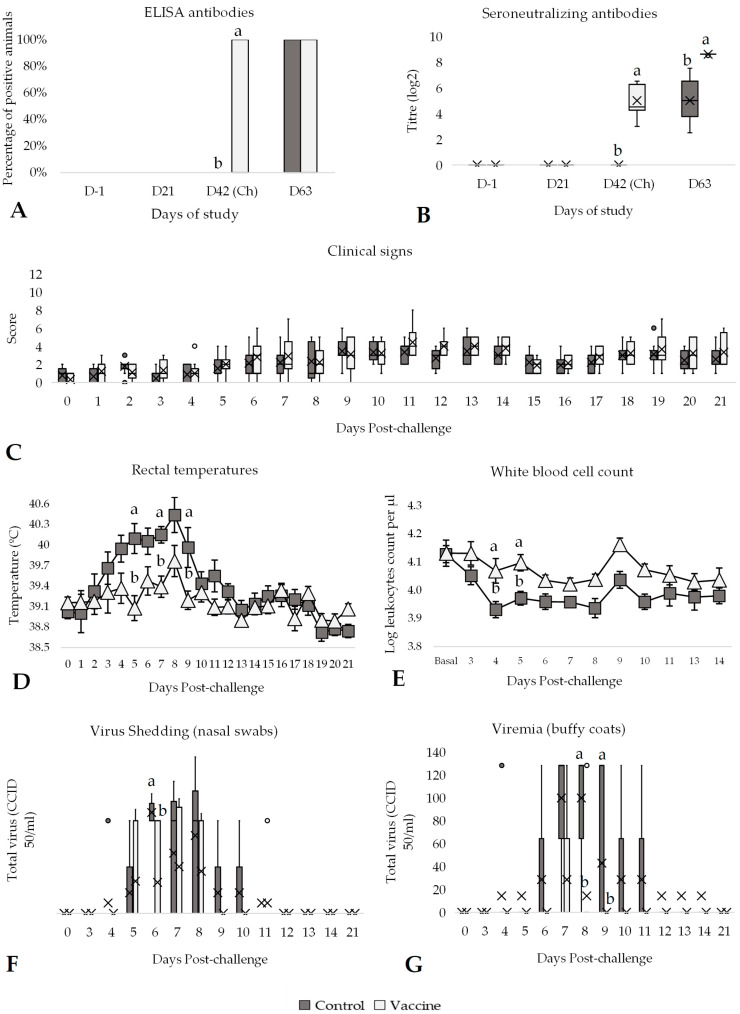
Efficacy of the DIVENCE^®^ PENTA vaccine against a BVDV-2 challenge. Control: 9 animals, Vaccinated: 9 animals. (**A**) Percentage of animals with positive ELISA antibodies per group from day 0 to 63 of study (bars; Fisher’s exact test). (**B**) Seroneutralising antibodies per group measured by serum neutralisation from day 0 to 63 of study (boxplot log2 values; Mann–Whitney U test). (**C**) Daily scoring of clinical signs per group from day of challenge until 21 days post-challenge (boxplot; Mann–Whitney U test). (**D**) Daily rectal temperatures per group from day of challenge until 21 days after challenge (mean ± SE; *t* Test). (**E**) White blood cell counts per group from basal (average of day −1 and day of challenge) until 14 days after challenge (log mean ± SE; *t* Test). (**F**) Virus shedding per group detected in nasal swabs by PCR from day of challenge until 21 days after challenge (boxplot; Mann–Whitney U test). (**G**) Viraemia per group detected in buffy coats by PCR from day of challenge until 21 days after challenge (boxplot; Mann–Whitney U test). ^a,b^ Different superscripts indicate statistical differences between control and vaccinated groups (*p* < 0.05). Ch, challenge.

On the challenge day (42 days post-vaccination), 100% of the vaccinated animals tested positive by both ELISA and SN, whereas the control group remained negative before challenge (Figure 3A,B). Furthermore, the average antibody titres (SN) in the vaccinated group were significantly higher (*p* < 0.05) than those in the control group (Figure 3B). After the challenge, all animals (vaccinated and control) had antibodies against BVDV, but the SN antibody titres in the vaccinated group remained significantly higher (*p* < 0.05) compared to the control group (Figure 3B). In terms of clinical signs, no significant differences were observed between the groups during the post-challenge period (Figure 3C, Supplementary Table S2).

Subsequently to the BVDV-2 challenge, a peak of rectal temperature was noticed from days 3 to 9 post-challenge, where the average of rectal temperatures in the control group (40.04 ± 0.08 °C) was significantly higher (*p* < 0.05) than in the vaccinated group (39.37 ± 0.15; Supplementary Table S2). At day 5, 7 and 9, these differences were statistically significant (*p* < 0.05) between the control and vaccinated groups (Figure 3D). The control group had significant (*p* < 0.05) hyperthermia (8.33 ± 1.27 days) for an additional 4 days compared to the vaccinated group (4.44 ± 1.25 days; Supplementary Table S2). Moreover, the average WBC count was significantly lower in the control animals (*p* < 0.05) compared to the vaccinated animals on days 4 and 5 post-challenge (Figure 3E). The average WBC count observed between the vaccinated (4.03 ± 0.02 logWBC counts) and control group (3.93 ± 0.02) during the post-challenge period was also significantly different (*p* < 0.05; Supplementary Table S2). Furthermore, a significant reduction in WBC counts between the basal day and day 4 post-challenge was observed in the control group (*p* < 0.01), whereas no significant change was detected in the vaccinated group over the same period (*p* = 0.980).

All the nasal and buffy coat samples during the vaccination period until the challenge day were negative by PCR (Figure 3F,G). From days 4 to 12 post-challenge, several animals excreted BVDV-2 in nasal swab samples. The virus shedding was significantly higher (*p* < 0.05) in the control group compared to the vaccinated group on day 6 post-challenge (day 48 of the study; Figure 3F). Moreover, the percentage of animals shedding BVDV-2 on day 6 was 67% significantly (*p* < 0.05) lower in the vaccinated group compared to the control group. Considering the entire post-challenge period, the average total virus shedding from nasal swabs in the control group (6.81 ± 2.60 CCID_50_/mL) was significantly higher (*p* < 0.05) compared to the vaccinated group (2.77 ± 1.32 CCID_50_/mL; Supplementary Table S2). Similarly, the virus titration from buffy coat samples of the control group was significantly (*p* < 0.05) higher than in the vaccinated group 8 and 9 days post-challenge (Figure 3G). The total virus titre from buffy coat samples was also significantly (*p* < 0.05) higher in the control group (36.20 ± 11.71 CCID_50_/mL) compared to the vaccinated group (3.88 ± 11.71 CCID_50_/mL) during the post-challenge period, as well as the days with viraemia (3.11 days vs. 0.33 days, respectively; Supplementary Table S2).

### 3.3. Efficacy Against IBR Challenge

**Figure 4:**
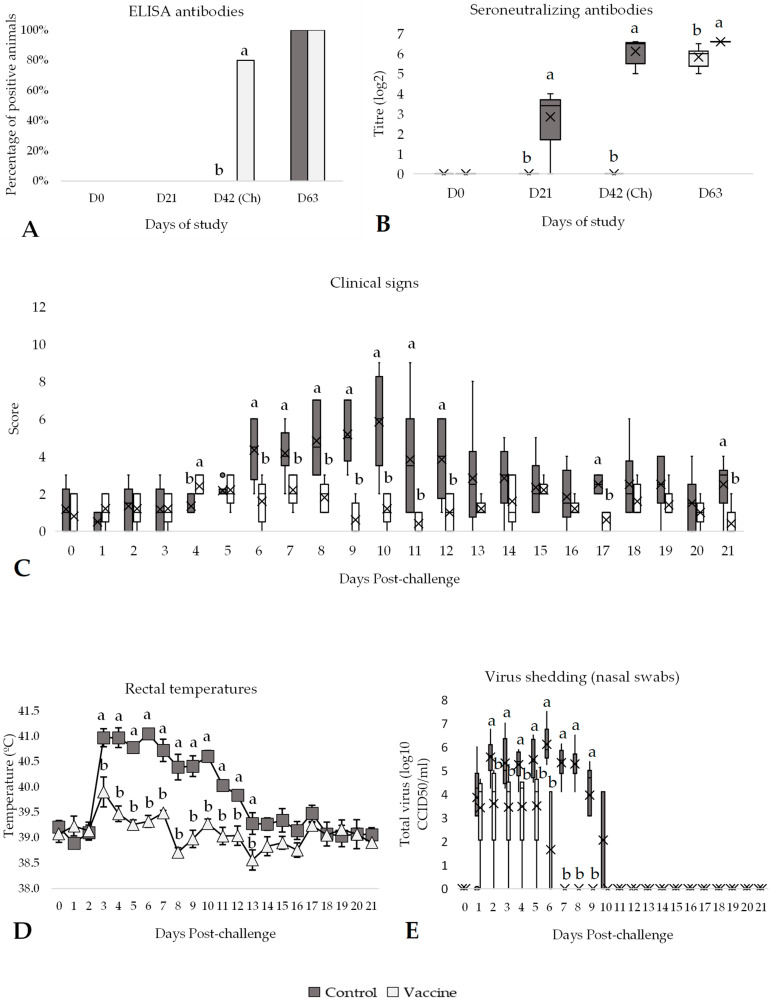
Efficacy of the DIVENCE^®^ PENTA vaccine against an IBR challenge. Control: 6 animals, Vaccinated: 6 animals. (**A**) Percentage of animals with positive ELISA antibodies per group from day 0 to 63 of study (bars; Fisher’s exact test). (**B**) Seroneutralising antibodies per group measured by serum neutralisation assay from days 0 to 63 of the study (boxplot log2 values; Mann–Whitney U test). (**C**) Daily scoring of clinical signs per group from day of challenge until 21 days post-challenge (boxplot; Mann–Whitney U test). (**D**) Daily rectal temperatures per group from day of challenge until 21 days after challenge (mean ± SE; *t* test). (**E**) Virus shedding per group detected in nasal swabs by cell culture from day of challenge until 21 days after challenge (boxplot log10 values; Mann–Whitney U test). ^a,b^ Different superscripts indicate statistical differences between control and vaccinated groups (*p* < 0.05). Ch, challenge.

At day 42 of the study (day of challenge), all vaccinated animals had seroneutralizing antibodies and 4 out of 5 had positive ELISA antibodies, a significantly higher (*p* < 0.05) percentage compared to control animals (Figure 4A,B). Meanwhile, all control animals remained negative until challenge day. Then, the SN antibody titres of vaccinated animals were significantly higher (*p* < 0.05) compared to control animals (Figure 4B). After challenge, all animals were positive for both ELISA and SN antibody titres, which were significantly higher (*p* < 0.05) in the vaccinated group compared to the control animals (Figure 4B). After the challenge, all control animals developed clinical signs associated with IBR infection. Specifically, from days 6 to 12 post-challenge, the clinical signs score was significantly higher (*p* < 0.05) in the control group compared to the vaccinated group (Figure 4C). In this regard, control animals showed nasal discharge, ocular discharge, depression, and dyspnoea, whereas vaccinated animals only presented mild clinical signs after IBR infection, mainly nasal discharge, and ocular discharge. Consequently, for the entire post-challenge period, the score in the control group (median of 2) was significantly higher (*p* < 0.05) than the average score in the vaccinated group (median of 1; Supplementary Table S3).

In addition, all control animals showed significantly higher rectal temperatures for 3 to 13 days post-challenge compared to vaccinated animals (>40 °C, *p* < 0.05; Figure 4D). From day 6 to day 9 post-challenge, the control group consistently showed rectal temperatures that were, on average, 1.27 °C significantly higher than those of the vaccinated group (*p* < 0.05). Furthermore, the control group showed significantly more days with hyperthermia, with 9 additional days over 39.5 °C compared to the vaccinated group (*p* < 0.05). Specifically, the control group has an average rectal temperature of 40.46 ± 0.12 °C and 12.17 ± 1.3 days over 39.5 °C, whereas the vaccinated group had an average of 39.19 ± 0.08 °C and 3.00 ± 1.3 days over 39.5 °C; Supplementary Table S3).

Virus isolated from nasal swabs on days 2 to 9 post-challenge was significantly higher (*p* < 0.05) in the control group compared to the vaccinated group (Figure 4E). Al-though all animals, both controls and vaccinated, shed virus over several days post-challenge, the animals in the control group showed a significantly higher (*p* < 0.05) amount of the IBR virus compared to the vaccinated animals and for nearly 5 additional days (9.17 days vs. 4.40 days, respectively; *p* < 0.05) (Supplementary Table S3).

### 3.4. Efficacy Against BRSV Challenge

**Figure 5:**
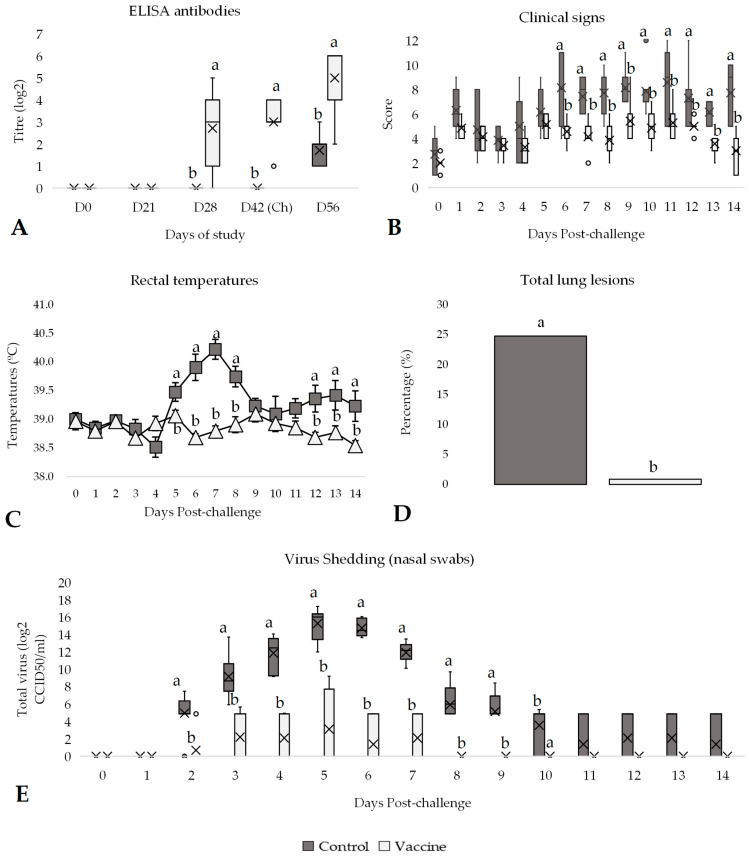
Efficacy of the DIVENCE^®^ PENTA vaccine against BRSV challenge. Control: 7 animals, Vaccinated: 7 animals. (**A**) ELISA antibodies response per group from day 0 to 56 of study (boxplot log2 values; Mann–Whitney U test). (**B**) Daily scoring of clinical signs per group from day of challenge until 14 days after challenge (boxplot; Mann–Whitney U test). (**C**) Daily rectal temperatures per group from day of challenge until 14 days after challenge (mean ± SE; *t* Test). (**D**) Total lung lesions per group 14 days after challenge (%; *t* Test). (**E**) Virus shedding per group detected in nasal swabs by PCR, from day of challenge until 14 days after challenge (boxplot log2 values; Mann–Whitney U test). ^a,b^ Different superscripts indicate statistical differences between control and vaccinated groups (*p* < 0.05). Ch, challenge.

All animals were sampled before vaccination and remained negative for ELISA antibodies until the challenge day, except for the vaccinated group. On day 28 of the study (the second vaccination), the percentage of positive animals in the vaccinated group increased significantly (*p* < 0.05) compared to the control group, as nearly 86% of the vaccinated animals developed antibodies against BRSV (Figure 5A). After the challenge, all animals became seropositive, but the antibody titre of the vaccinated group was significantly (*p* < 0.05) higher compared to the control group (Figure 5A). General clinical signs were also monitored throughout the entire study, with no alterations observed in relation to vaccination during the vaccination phase. After the BRSV challenge, the control group showed an increase in the score, especially from day 5 to day 12 (Figure 5B). Hence, a notably significant reduction (*p* < 0.05) in clinical signs was observed after challenge in the vaccinated animals compared to the control animals (median of 6.5 vs. 4, respectively; Supplementary Table S4).

In reference to rectal temperatures, as observed in clinical signs, a higher significant increase in the control animals was observed from day 5 to day 8 post-challenge compared to vaccinated animals (Figure 5C). Consequently, the overall rectal temperature post-challenge was almost 1 °C significantly higher (*p* < 0.05) in the control group (39.84 ± 0.13 °C) compared to the vaccinated group (38.86 ± 0.06 °C), as well as the number of days with hyperthermia (4.43 ± 0.9 vs. 0.57 ± 0.3 days over 39.5 °C, *p* < 0.05; Supplementary Table S4). Furthermore, a significant increase in rectal temperature between the basal day and day 7 post-challenge was observed in the control group (*p* < 0.01), whereas no significant change was detected in the vaccinated group over the same period (*p* = 0.998). The results also reflected statistically significant differences in lung affections between groups, with control animals showing evident lung lesions, while vaccinated animals’ lesions were practically non-existent. Specifically, the total lung lesion percentage was significantly higher in the control group (24.76%) compared to the vaccinated group (0.87%, *p* < 0.05; Figure 5D).

All animals were sampled weekly using nasal swab from the first vaccination dose until the end of the challenge. BRSV was not detected in any of the samples collected before the challenge. On the first day post-challenge, BRSV was still not detected in any sample. However, from two days post-challenge until the end of the study, BRSV was detected in several samples. The control group had significantly higher (*p* < 0.05) virus excretion, particularly from 2 to 10 days post-challenge, compared to the vaccinated group (Figure 5E). These differences in excretion between vaccinated and control groups were also observed by analysing the average of total virus excretion per group during the entire post-challenge period (from days 1 to 14 after challenge; Supplementary Table S4). Vaccinated animals had significantly (*p* < 0.05) lower total virus excretion compared to control animals (0.89 vs. 6.86 log2 CCID_50_/mL, respectively) and number of days shedding (2.14 days vs. 9.71 days, respectively).

### 3.5. Efficacy Against PI-3 Challenge

**Figure 6:**
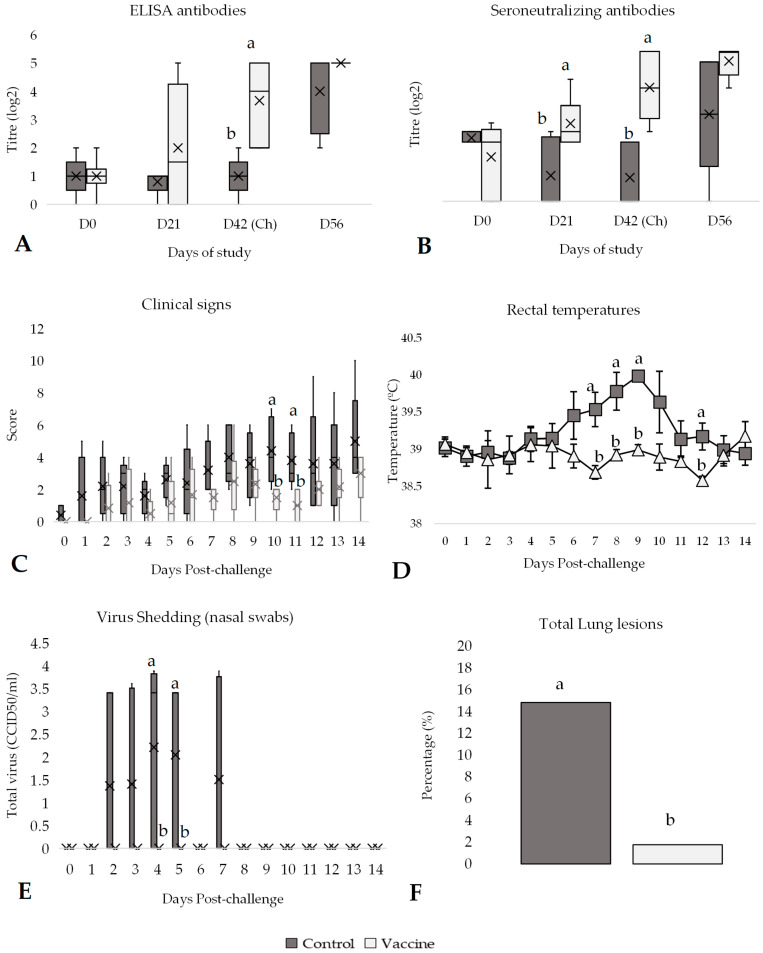
Efficacy of the DIVENCE^®^ PENTA vaccine against a PI-3 challenge. Control: 6 animals, Vaccinated: 5 animals. (**A**) ELISA antibodies response per group from day 0 to 56 of study (boxplot log2 values; Mann–Whitney U test). (**B**) Seroneutralising antibodies per group measured by serum neutralisation assay from day 0 to 56 of study (boxplot log2 values; Mann–Whitney U test). (**C**) Daily scoring of clinical signs per group from day of challenge until 14 days after challenge (boxplot; Mann–Whitney U test). (**D**) Daily rectal temperatures per group from day of challenge until 14 days after challenge (mean ± SE; *t* test). (**E**) Virus shedding per group detected in nasal swabs by cell culture, from day of challenge until 14 days after challenge (boxplot; Mann–Whitney U test). (**F**) Total lung lesions per group 14 days after challenge (% mean ± SE; *t* test). ^a,b^ Different superscripts indicate statistical differences between control and vaccinated groups (*p* < 0.05). Ch, challenge.

All the animals enrolled in the study had low antibody titres against PI-3 (<45 M/P values, Figure 6A) measured by ELISA on day 0 of the study (day of vaccination). After challenge, vaccinated animals had significantly higher (*p* < 0.05) average antibody titres compared to the control animals (Figure 6A). Similarly, on day 42 of the study, the vaccinated group had a significantly higher (*p* < 0.05) average SN antibody titre compared to the control group (Figure 6B). After the challenge, the antibody titres (ELISA and SN) increased in both groups. Similarly, the control group had higher clinical signs scores compared to the vaccinated group after challenge. These differences were statistically significant (*p* < 0.05) on days 10 and 11 post-challenge (Figure 6C).

In addition, all animals had rectal temperatures below 39.5 °C before the challenge. After challenge, a peak of rectal temperatures was observed between days 6 and 10, where the average of rectal temperatures in the control group was significantly higher (*p* < 0.05) than in the vaccinated group (Figure 6D). During post-challenge, all control animals had hyperthermia, whereas only 2 vaccinated animals presented fever. Thus, the average rectal temperature was significantly higher (*p* < 0.05) in the control group (39.68 ± 0.68 °C) compared to the vaccinated group (38.89 ± 0.05 °C; Supplementary Table S5).

After the challenge, nasal swabs were obtained every day until day 14 post-challenge to detect PI-3 virus shedding. From the beginning of the study (day 0) to the first day post-challenge, PI-3 was still not detected in any sample. However, from day 2 to 7 post-challenge, PI-3 was detected in several samples of the control group. None of the nasal swab samples collected from vaccinated animals contained PI-3 throughout the entire study (Figure 6E). The control group had higher virus shedding mainly from day 2 to 7 (except on day 6) post-challenge. Significant differences (*p* < 0.05) were observed between vaccinated and control groups on days 4 and 5 post-challenge (Figure 6E). The difference in virus shedding and the number of days shedding between vaccinated and control groups was also compared throughout the entire post-challenge period. Vaccinated animals had significantly lower (*p* < 0.05) total virus shedding (0.0 ± 0.0 log2 CCID_50_/mL) compared to control animals (1.42 ± 0.48 log2 CCID_50_/mL; Supplementary Table S5) and number of days shedding (0 days vs. 2.4 days, respectively; Supplementary Table S5), as none of the vaccinated animals excreted PI-3 post-challenge. Moreover, the average of the total lung affection in vaccinated animals was significantly lower (*p* < 0.05) than in the control group (1.78% vs. 14.84%, respectively, *p* < 0.05; Figure 6F).

## 4. Discussion

The currently available multivalent BRD vaccines against BVDV types 1 and 2, IBR, BRSV and PI-3 have been shown to induce strong serological responses against these viral pathogens and to reduce disease in calves [11,12,22]. The findings of the present study align with previous research [13,17,18,19], providing compelling evidence of a significant reduction in BRD in calves vaccinated with DIVENCE^®^ PENTA. Animals that were vaccinated showed high humoral immune response, fewer clinical symptoms, lower rectal temperatures, no affection of leukocyte counts, reduced viral load and duration, and milder pulmonary lesions compared to the control animals. These results reinforce the efficacy of the multiviral vaccine in preventing young calves from contracting BVDV types 1 and 2, IBR, BRSV and PI-3.

In the present study, the serum and neutralizing antibody responses in vaccinated calves increased after the initial vaccination for all viruses, with onset occurring no later than 3 weeks after. Nearly 100% of the calves had seroconverted by the day of the challenge, consistent with findings from other studies using live vaccines [11,13,23,24]. Although antibody levels are not a direct measure of vaccine efficacy, they can play a critical role in mitigating the severity of symptoms associated with BRD (i.e., milder clinical signs). Moreover, no decrease in WBC counts was observed during the post-challenge period in the vaccinated group, demonstrating that vaccination with DIVENCE^®^ PENTA prevents immunosuppression [25]. Additionally, the results demonstrate the high efficacy of the vaccine in reducing hyperthermia following challenge in all cases, in terms of rectal temperature and duration.

In the case of BVDV challenges, the viruses were isolated either from nasal swabs or buffy coat samples from all control animals, confirming active infection. In contrast, only a few of the vaccinated animals showed viraemia, and their viral titres were notably low. This reduction is clinically relevant, as previous authors have demonstrated a link between the level of viraemia and the severity of the disease associating elevated viral titres with more severe clinical outcomes [26,27]. In our study, the vaccinated animals not only showed a reduction in viraemia levels but also showed a shortened duration of viraemia, contributing to a significant decrease in the total viral titre detected. This reduction in viral load consequently lowers the potential for virus spread, as reported by other authors [28], and in line with previous studies using the same vaccine [18].

Beyond the reduction in viral load, a key indicator of a vaccine’s efficacy and safety is its ability to prevent leukopenia during acute BVDV infections, because of its link with immunosuppression and increased susceptibility to secondary infections [25]. The calves in the present study immunized with the recombinant protein-based BVDV vaccine maintained the levels of WBC counts post-challenge following BVDV challenge, further supporting the immunological efficacy of DIVENCE^®^ PENTA. These results are consistent with previous findings in studies assessing WBC dynamics following administration of BVDV vaccines [29,30]. In particular, Loy et al. [30] showed a higher reduction in the degree of leukopenia following vaccination with high doses of recombinant BVDV vaccine in calves, highlighting their efficacy when properly administered in healthy animals.

Regarding the IBR challenge, both control and vaccinated animals shed virus. However, the vaccinated group demonstrated a shorter duration of viral excretion, with shedding reduced by 5 days and almost 1000-fold lower than those of the controls. This is consistent with findings from other authors, which have similarly reported a reduction in the duration of IBR viral shedding in vaccinated animals [31]. Furthermore, the results demonstrated a robust serological response to IBR, with no negative impact on seroconversion following vaccination, in agreement with previous studies [32,33]. Notably, vaccination with DIVENCE^®^ PENTA, containing a live gene-deleted BoHV-1 (gE/tk double deletion), also led to a strong reduction in hyperthermia and clinical signs following immunization. These results suggest that vaccination can effectively mitigate both the severity and the transmission potential of BVDV and IBR viral infections in cattle.

In the case of the BRSV challenge, the reduced viral load and lung lesions indicated that the vaccinated calves developed a protective immune response [34,35]. Some authors have also demonstrated that calves vaccinated with MLV BRSV vaccines exhibit significantly less severe pulmonary lesions compared to unvaccinated calves when challenged with the virus [13,36]. This reduction in lung damage is associated with enhanced mucosal immune responses, including increased levels of IgA antibodies in the respiratory tract. Additionally, it has been suggested that local immune responses, including IgA production and T-cell mediated mechanisms, are crucial in conferring protection against BRSV, and these responses can be efficiently primed by live vaccines [37]. Consequently, live BRSV vaccines not only help in mitigating the clinical severity of respiratory disease but, also, contribute to better lung health and reduced morbidity in cattle populations. Lung lesions were reduced from 24.76% in controls to 0.87% in vaccinated animals, corresponding to a 96% reduction. Notably, vaccination prevented hyperthermia in the vaccinees during the post-challenge period, further supporting the vaccine’s protective effect. In this study, all vaccinated animals developed an evident immune response following vaccination. Taken together, these findings underscore the value of live BRSV vaccines in mitigating clinical disease and limiting viral dissemination within herds.

A significant difference was also observed between the groups in response to the PI-3 challenge. None of the vaccinated animals presented viral shedding from day 0 to day 42 of the study, suggesting that all animals had no prior exposure to PI-3 before the challenge. This absence of PI-3 excretion remained in the vaccinated group throughout the entire infection phase, further supporting the protective efficacy of the vaccine as shedding of PI-3 was prevented in vaccinated animals. Additionally, the experimental challenge induced notable respiratory lung lesions in the control group, while the vaccinated group showed only mild or no lung lesions at all. Lung lesions were reduced from 14.84% in controls to 1.78% in vaccinated animals, corresponding to an 88% reduction. These findings align with previous research, which demonstrated a reduction in the risk of lung lesions associated with pneumonia following vaccination [38].

Together, these findings clearly demonstrate that vaccination with DIVENCE^®^ PENTA helps to prevent BRD and the associated costs of the disease, highlighting its role in reducing both the spread of infection and the risk of pneumonia. Importantly, DIVENCE^®^ PENTA combines recombinant antigens from both BVDV-1 and BVDV-2, providing broad-spectrum protection without compromising the host’s immune function. The absence of leukopenia observed throughout the study underscore its efficacy. The inclusion of a double-deleted marker IBR strain in the formulation further enhances the value of the vaccine in a field context. This marker deletion (gE gene) allows for serological differentiation between vaccinated and infected animals (DIVA strategy), while maintaining a solid safety profile, as widely documented in European regulatory data. Additionally, the E2-recombinant protein-based design of the BVDV vaccine component also enables differentiation of infected from vaccinated animals (DIVA), offering an extraordinary advantage for disease surveillance and compliance with international trade policies in beef and dairy markets. Nevertheless, although the number of animals per group was determined in accordance with European Pharmacopoeia requirements, larger-scale field studies will be required to confirm and further extend the present findings.

## 5. Conclusions

The multivalent vaccine DIVENCE^®^ PENTA, which targets BVDV types 1 and 2, IBR, BRSV, and PI-3, demonstrated a strong immunogenic effect, significantly increasing the humoral immune response no later than 21 days post-vaccination. Upon challenge, vaccinated animals exhibited a statistically significant reduction in hyperthermia, leukopenia, viral shedding, duration of viremia, and lung lesions.

Importantly, these findings complement previous work showing that DIVENCE^®^ PENTA also provides field efficacy under commercial fattening conditions and foetal protection against BVDV, thereby extending its utility across multiple production stages. Taken together, the evidence supports the use of DIVENCE ^®^ PENTA as a comprehensive tool for the prevention of BRD, offering broad-spectrum protection from early calfhood through the fattening phase, and contributing to healthier herds and reduced economic losses against the major viral pathogens involved in this complex.

## 6. Patents

A patent application has been filed by HIPRA SCIENTIFIC S.L.U. for vaccines against bovine viral diarrhoea virus and bovine respiratory disease, including the novel vaccine DIVENCE^®^. M. Gibert is one of the inventors of said patent application.

## Data Availability

The data presented in this study are available from the corresponding author upon reasonable request.

## Supplementary Materials

The following supporting information can be downloaded at: https://www.mdpi.com/article/10.3390/vaccines13100999/s1, Table S1: Efficacy parameters measured per group (mean ± SE) post-challenge against BVDV-1; Table S2: Efficacy parameters measured per group (mean ± SE) post-challenge against BVDV-2; Table S3: Efficacy parameters measured per group (mean ± SE) post-challenge against IBR; Table S4: Efficacy parameters measured per group (mean ± SE) post-challenge against BRSV; Table S5: Efficacy parameters measured per group (mean ± SE) post-challenge against PI-3.

## Abbreviations

The following abbreviations are used in this manuscript:

BoHV-1	Bovine herpesvirus type 1
BRD	Bovine respiratory disease
BRSV	Bovine respiratory syncytial virus
BVDV	Bovine viral diarrhoea virus
DIVA	Differentiating infected from vaccinated animals
ELISA	Enzyme-linked immunosorbent assay
gE	Glycoprotein E
IBR	Infectious bovine rhinotracheitis
IgA	Immunoglobulin A
MLV	Modified-live vaccine
PBS	Phosphate-buffered saline
PI-3	Bovine parainfluenza-3 virus
RT-qPCR	Reverse-transcription quantitative PCR
SN	Serum-neutralisation (antibody) test
WBC	White blood cell
