# Supplementary material for: Respiratory Efficacy of a Multivalent Marker Vaccine Against Bovine Viral Diarrhoea Virus Types 1 and 2, Infectious Bovine Rhinotracheitis Virus, Bovine Respiratory Syncytial Virus, and Bovine Parainfluenza-3 Virus in Young Calves
Source: Vaccines (Basel). 2025 Sep 24;13(10):999. doi: 10.3390/vaccines13100999 (PMC12568246; doi:10.3390/vaccines13100999)
Supplement: Supplementary file 1 [file vaccines-13-00999-s001.zip › vaccines-3795918-supplementary.pdf]

Table S1. Efficacy parameters measured per group (mean ± SE) post-challenge against BVDV-1.

Parameters	Control	Vaccinated	Statistical test
Clinical signs scores PCh*	0[a]	0[a]	Mann-Whitney U test
Rectal temperatures (ºC, D6 to D9 PCh)	39.60 ± 0.09[a]	39.27 ± 0.10[b]	*t* test
Days with hyperthermia PCh (>39.5ºC)	2.50 ± 0.05[a]	1.13 ± 0.02[b]	*t* test
White blood cell count PCh (logWBC counts, D3 to D9 PCh)	3.82 ± 0.03[a]	3.88 ± 0.02[a]	*t* test
Total virus shedding PCh ($CCID_{50}$/ml, nasal swabs, D3 to D8)	28.88 ± 5.20[a]	12.92 ± 1.60[a]	*t* test
Number of days shedding PCh (nasal swabs)	4.38 ± 0.50[a]	3.50 ± 0.60[a]	*t* test
Total viremia PCh ($CCID_{50}$/ml, buffy coats, D3 to D7 PCh)	62.80 ± 5.54[a]	7.85 ± 1.71[b]	Mann-Whitney U test
Number of days shedding PCh (buffy coats)	2.00 ± 0.46[a]	0.25 ± 0.16[b]	Mann-Whitney U test

*The median value (from D1 to D21 post-challenge) per group is presented in this parameter (clinical signs). [a,b]Statistical differences between control and vaccinated groups ($p < 0.05$). Pch = Post-challenge (day 1 to 21); D = days

Table S2. Efficacy parameters measured per group (mean ± SE) post-challenge against BVDV-2.

Parameters	Control	Vaccinated	Statistical test
Clinical signs scores PCh*	2[a]	2.5[a]	Mann-Whitney U test
Rectal temperatures (ºC, D6 to D9 PCh)	40.04 ± 0.08[a]	39.37 ± 0.15[b]	*t* test
Days with hyperthermia PCh (>39.5ºC)	8.33 ± 1.27[a]	4.44 ± 1.25[b]	*t* test
White blood cell count PCh (logWBC counts, D3 to D10 PCh)	3.93 ± 0.02[a]	4.03 ± 0.02[b]	*t* test
Total virus shedding PCh ($CCID_{50}$/ml, nasal swabs D3 to D11 PCh)	6.81 ± 2.60[a]	2.77 ± 1.32[b]	*t* test
Number of days shedding PCh (nasal swabs)	3.11 ± 0.35[a]	1.67 ± 0.47[b]	*t* test
Total viremia PCh ($CCID_{50}$/ml, buffy coats D3 to D13 PCh)	36.20 ± 11.71[a]	3.88 ± 1.94[b]	Mann-Whitney U test
Number of days shedding PCh (buffy coats)	3.11 ± 1.0[a]	0.33 ± 0.17[b]	Mann-Whitney U test

*The median value (from D1 to D21 post-challenge) per group is presented in this parameter (clinical signs). [a,b]Statistical differences between control and vaccinated groups ($p < 0.05$). Pch = Post-challenge (day 1 to 21); D = days.

Table S3. Efficacy parameters measured per group (mean ± SE) post-challenge against IBR.

Parameters	Control	Vaccinated	Statistical test
Clinical signs scores PCh*	2[a]	1[b]	Mann-Whitney U test
Rectal temperatures (ºC, D3 to D13 PCh)	40.46 ± 0.12[a]	39.19 ± 0.08[b]	*t* test
Days with hyperthermia PCh (>39.5ºC)	12.17 ± 1.30[a]	3.00 ± 1.26[b]	*t* test
Total virus shedding PCh (log10 $CCID_{50}$/ml, nasal swabs D1 to D10)	4.81 ± 0.21[a]	1.90 ± 0.49[b]	*t* test
Number of days shedding PCh (nasal swabs)	9.17 ± 0.31[a]	4.40 ± 1.12[b]	Mann-Whitney U test

*The median value (from D1 to D21 post-challenge) per group is presented in this parameter (clinical signs). [a,b]Statistical differences between control and vaccinated groups ($p < 0.05$). Pch = Post-challenge (day 1 to 21); D = days

Table S4. Efficacy parameters measured per group (mean ± SE) post-challenge against BRSV.

Parameters	Control	Vaccinated	Statistical test
Clinical signs scores PCh*	6.5[a]	4[b]	Mann-Whitney U test
Rectal temperatures (ºC, D5 to D8 PCh)	39.84 ± 0.13[a]	38.86 ± 0.06[b]	*t* test
Days with hyperthermia PCh (>39.5ºC)	4.43 ± 0.90[a]	0.57 ± 0.30[b]	Mann-Whitney U test
Total virus shedding PCh (log2 $CCID_{50}$/ml, nasal swabs D2 to D14)	6.86 ± 0.47[a]	0.89 ± 0.29[b]	*t* test
Number of days shedding PCh (nasal swabs)	9.71 ± 0.87[a]	2.14 ± 0.67[b]	*t* test

*The median value (from D1 to D14 post-challenge) per group is presented in this parameter (clinical signs).
[a,b]Statistical differences between control and vaccinated groups ($p < 0.05$). Pch = Post-challenge (day 1 to 14);
D = days

Table S5. Efficacy parameters measured per group (mean ± SE) post-challenge against PI-3.

Parameters	Control	Vaccinated	Statistical test
Clinical signs scores PCh*	2.5[a]	1.5[a]	Mann-Whitney U test
Rectal temperatures (ºC, D6 to D10 PCh)	39.68 ± 0.12[a]	38.89 ± 0.05[b]	*t* test
Days with hyperthermia PCh (>39.5ºC)	3.60 ± 0.68[a]	1.17 ± 0.75[a]	Mann-Whitney U test
Total virus shedding PCh (log 2 $CCID_{50}$/ml, nasal swabs)	1.42 ± 0.48[a]	0.00 ± 0.00[b]	Mann-Whitney U test
Number of days shedding PCh (nasal swabs)	2.40 ± 0.81[a]	0.00 ± 0.00[b]	Mann-Whitney U test

*The median value (from D1 to D14 post-challenge) per group is presented in this parameter (clinical signs).
[a,b]Statistical differences between control and vaccinated groups ($p < 0.05$). Pch = Post-challenge (day 1 to 14);
D = days
